# Depression and/or PTSD Comorbidity Affects Response to Antidepressants in Those With Alcohol Use Disorder

**DOI:** 10.3389/fpsyt.2021.768318

**Published:** 2022-01-04

**Authors:** Peter J. Na, Elizabeth Ralevski, Oluwole Jegede, Aaron Wolfgang, Ismene L. Petrakis

**Affiliations:** ^1^Department of Psychiatry, VA Connecticut Healthcare System, West Haven, CT, United States; ^2^Department of Psychiatry, School of Medicine, Yale University, New Haven, CT, United States

**Keywords:** alcohol use disorder, major depressive disorder, PTSD, desipramine, noradrenergic, paroxetine, SSRI, naltrexone

## Abstract

**Objective:** Depression and post-traumatic stress disorder (PTSD) highly co-occur with alcohol use disorder (AUD). The comparative effects of noradrenergic vs. serotonergic antidepressants on drinking and depressive outcomes for those with AUD and co-occurring depression and/or PTSD are not well known.

**Methods:** This study was an analysis of a randomized control trial of 128 patients with AUD who had co-occurring depression and/or PTSD. They were randomized to treatment with paroxetine vs. desipramine and naltrexone vs. placebo leading to four groups: paroxetine plus naltrexone, paroxetine plus placebo, desipramine plus naltrexone, and desipramine plus placebo. Outcomes were percent of drinking days, percent heavy drinking days, drinks per drinking day (Time Line Follow-back Method), and depressive symptoms (Hamilton Depression Scale). Groups compared were (1) depression without PTSD (depression group; *n* = 35), (2) PTSD without depression (PTSD group; *n* = 33), and (3) both depression and PTSD (comorbid group; *n* = 60).

**Results:** There were no overall significant differences in drinking outcomes by medication in the entire sample, and no significant interaction when diagnostic groups were not considered. However, when diagnostic groups were included in the model, the interactions between time, diagnostic group, and medication (desipramine vs. paroxetine) were significant for percent drinking days (*p* = 0.042), and percent heavy drinking days (*p* = 0.036); paroxetine showed better drinking outcomes within the depression group, whereas desipramine showed better drinking outcomes in the PTSD and comorbid groups. Regarding depressive symptoms, paroxetine was statistically superior to desipramine in the total sample (*p* = 0.007), but there was no significant interaction of diagnostic group and medication. Naltrexone led to a decrease in craving but no change in drinking outcomes.

**Conclusions:** The results of this study suggest that drinking outcomes may respond differently to desipramine and paroxetine depending on comorbid MDD and/or PTSD.

## Introduction

Alcohol use disorder (AUD) is a common condition with a lifetime prevalence of approximately 30% in the general population (1) and highly prevalent in veterans (2). Among adults with AUD, 30–59% also meet criteria for post-traumatic stress disorder (PTSD) (3), and up to 50% meet criteria for major depressive disorder (MDD) (4). Among veterans with AUD, there are also high rates of comorbid psychiatric conditions, including MDD and PTSD (5, 6). Comorbidity between MDD and PTSD is also common; one recent study reported that 36.8% of veterans with co-occurring PTSD and AUD also screened positive for MDD (7).

Alcohol use disorder and co-occurring MDD or PTSD is associated with more severe symptoms and worse alcohol-related outcomes, functional impairment, and increased risk of suicide (7–10). However, to date, very few studies have examined the effectiveness of pharmacologic treatments when there are multiple psychiatric diagnoses, such as co-occurring MDD and/or PTSD in those with AUD. When comorbid conditions occur, clinically it is often unclear whether treatments which have been developed in individuals without the comorbid condition are effective in “real world” clinical settings where comorbid conditions are often the rule rather than the exception (11).

Selective serotonin reuptake inhibitors (SSRIs), including paroxetine, are first-line evidence-based pharmacotherapies for both PTSD and depression (12, 13). The literature evaluating the effectiveness of SSRIs when used in individuals with comorbid AUD is mixed. Studies mostly support the use of SSRIs in reduction of PTSD and depressive symptoms in patients who have AUD with co-occurring PTSD (14, 15) and MDD (16, 17), respectively. However, most studies do not support the role of SSRIs in improving alcohol use outcomes in patients with co-occurring PTSD and AUD (15, 18–22) and MDD and AUD (20, 23).

Tricyclic antidepressants with noradrenergic reuptake inhibition (NRI) properties were one of the first medications for PTSD supported by double-blind, placebo-controlled clinical trials (24). Desipramine is an oral tricyclic antidepressant, and has both noradrenergic and serotonergic reuptake inhibiting properties (25). Noradrenergic mechanisms are implicated in the pathophysiology of PTSD (26) and depression (27). While noradrenergic antidepressants are approved for treatment of depression (28), they have not been rigorously tested in PTSD (14). Few studies have directly compared them to SSRIs (29) and their role in comorbidity is not well-known. Our work comparing the noradrenergic antidepressant desipramine to the SSRI paroxetine in 88 individuals with co-occurring PTSD and AUD found that desipramine and paroxetine were equivalent in decreasing symptoms of PTSD, but desipramine was superior to paroxetine in reducing alcohol consumption, with significantly reduced heavy drinking days and number of drinks per week compared to paroxetine (14). Depressive symptoms significantly decreased over time with no difference between treatment arms. Of note, adding naltrexone did not demonstrate any additional advantage on drinking outcomes although did significantly improve craving for alcohol. All in all, extant literature suggests that co-occurring psychiatric disorders in patients with AUD complicate treatment and deserves further investigation to guide clinical decision making. Further, while some studies have evaluated the effectiveness of treatment of AUD when it co-occurs with either PTSD or MDD, the comorbidity of all three disorders has not yet been examined.

In this study, 128 subjects who had alcohol dependence [using the Diagnostic and Statistical Manual of Mental Disorders, Fourth Edition (DSM-IV)] who also had co-occurring PTSD and/or MDD were recruited to participate in a study comparing the effects of desipramine vs. paroxetine with or without naltrexone vs. placebo on alcohol use craving, mood, and PTSD symptoms. As noted above, we have previously published the results of those with PTSD with and without MDD (14). In a further effort to investigate the differences in treatment outcomes by comorbidity, we first compared the effects of desipramine vs. paroxetine and naltrexone vs. placebo regardless of diagnostic group in the full sample. Then we examined separately the groups with (1) current MDD alone (MDD group), (2) current PTSD alone (PTSD group), and (3) both MDD and PTSD (comorbid group), and compared alcohol use craving, as well as depressive symptoms by medication. The aim of the current study was to elucidate the role of comorbid diagnoses in treatment outcomes that may offer guidance in clinical settings.

## Materials and Methods

### Overview of the Parent Trial

**Figure 1:**
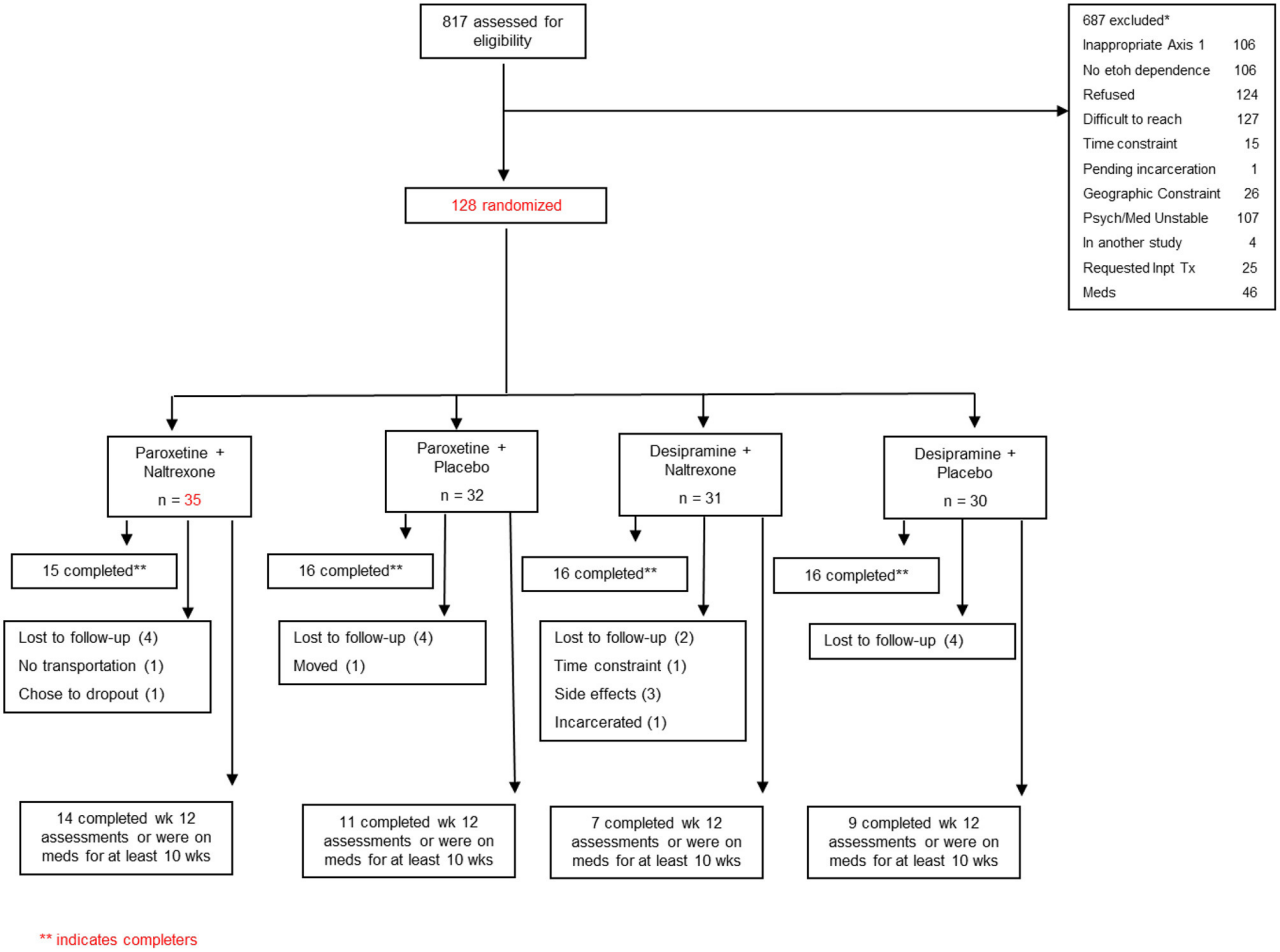
Flowchart describing the screening and randomization distribution for the participants in the study.

This 12-week multisite randomized controlled trial (RCT) of patients with AUD compared the effectiveness of desipramine vs. paroxetine with or without naltrexone vs. placebo. Full details of the study methodology have been described previously (14). Briefly, 128 patients with AUD were recruited from the outpatient clinics of the VA Connecticut Healthcare System (West Haven, CT), and the Bedford VA Medical Center (Bedford, MA). Participants who met criteria for current DSM-IV criteria for AUD and who used alcohol within the past 30 days and who had either current MDD or PTSD were included. Those with unstable psychotic symptoms or serious current psychiatric symptoms, such as suicidal or homicidal ideation, or medical conditions that would contraindicate the use of naltrexone, desipramine, or paroxetine were excluded. Participants could not be taking medications thought to affect alcohol consumption (e.g., naltrexone, disulfiram, or acamprosate), and were required to be abstinent for 2 days prior to randomization (see Figure 1). Yale Human Investigations Committee approved the study (West Haven VA IRB, as well as Bedford VA IRB). All participants provided written informed consent prior to enrollment.

Eligible participants were randomized to: (1) paroxetine plus naltrexone, (2) paroxetine plus placebo, (3) desipramine plus naltrexone, and (4) desipramine plus placebo. Desipramine was started at 25 mg per day and was gradually increased over 3 weeks to 200 mg per day. Paroxetine was started at 10 mg per day and gradually increased over 2 weeks to 40 mg per day. Naltrexone was started at 25 mg the first day, and 50 mg the following day and for the rest of the treatment. Medication adherence was assessed at every visit. Participants were provided Clinical Management/Compliance Enhancement therapy (30) administered by trained research personnel.

### Measures

#### Demographic Characteristics

Study participants reported their age, gender, marital status, and ethnicity.

#### Alcohol Use

Baseline severity of alcohol use was assessed with the Alcohol Dependence Scale (ADS) (31). A highly trained research personnel obtained a detailed self-report of daily alcohol and other substance use throughout the 12-week treatment period, as well as for 90 days prior to randomization using the Timeline Follow-back Method (TLFB) (32). Craving was measured at baseline and weekly throughout the study using the Obsessive Compulsive Drinking and Abstinence Scale (OCDS) (33).

#### Depression Assessment

Major depressive disorder diagnosis was made by a clinician-administered structured interview based on the DSM-IV (34). Depressive symptoms were assessed with the Hamilton Depression Rating Scale (HAM-D), a 17-item clinician administrated assessment scale that measures the severity of depressive symptoms over the past week with a range from 0 to 52 points (35).

#### PTSD Assessment

Post-traumatic stress disorder diagnosis was made by the clinician administered PTSD scale for DSM-IV (CAPS) (36). Please note that results regarding PTSD symptoms were reported in our parent study (14) since the CAPS were not administered to the entire sample.

### Statistical Analysis

Descriptive statistics were used to summarize the data on all randomized subjects. All continuous variables were examined for normal distribution using normal probability plots and Kolmogrorov-Smirnov tests. The alcohol use data was not normally distributed. Log transformations were applied, but normality was not achieved. Thus, the alcohol data was ranked and non-parametric tests for repeated measures analysis were used (37).

Baseline participant characteristics were summarized using frequency and proportion for categorical variables and mean and standard deviation for continuous variables. Baseline differences across diagnoses (MDD vs. PTSD vs. comorbid group) were compared using analyses of variance (ANOVA) or chi-squared test, as appropriate. The analyses were conducted on the intent-to-treat sample. All statistical testing was at a two-tailed alpha level of 0.05.

The outcome variables included: (a) measures of alcohol consumption (percent of drinking days, percent heavy drinking days, and drinks per drinking days), (b) measures of craving (OCDS total scores), and (c) MDD symptoms (HAM-D total scores). Mixed-effects models were used to assess changes in alcohol consumption, craving, and MDD symptoms over time. To better understand the differences among the four medication groups, data were analyzed comparing desipramine to paroxetine, or naltrexone to placebo, and their interactions in separate models. The diagnostic group (MDD, PTSD, and combined group) was used as a between-subject factor in the models, and time (12 weeks, or baseline vs. overall drinking during entire treatment) was used as a within-subject factor. The use of the mixed-effects models approach for the analysis of our data has several specific advantages. Unlike traditional repeated measures analyses, mixed-effects models allow for different numbers of observations per subject, use of all available data on each subject, and are unaffected by randomly missing data. They also provide flexibility in modeling the correlation structure of the data (38).

## Results

### Demographic Characteristics

On average, the full sample was 47.89 years old (SD = 9.1; range 46.27–49.52); the majority were male (91.3%) and Caucasian (73.2%). Table 1 presents the demographic and clinical characteristics by diagnostic groups. There were no significant differences in age, gender, and marital status across groups.

**Table 1:** Demographic and clinical characteristics of the sample.

**Variables**	**Depression only *n* = 35**	**PTSD only *n* = 33**	**Depression and PTSD *n* = 60**	***X^**2**^, p***
**Age, mean (SD)**	50.0 (9.3)	46.8 (9.1)	46.5 (9.0)	
**Male gender**, ***n*** **(%)**	33 (94.2)	30 (90.9)	53 (88.3)	4.75, 0.31
**Female gender**, ***n*** **(%)**	2 (5.8)	3 (9.1)	7 (11.7)	
**Marital Status**, ***n*** **(%)**				
Single	11 (31.4)	6 (18.1)	18 (30.0)	9.47, 0.48
Married	3 (8.5)	4 (12.1)	7 (11.6)	
Separated	2 (5.7)	7 (21.2)	6 (10.0)	
Divorced	17 (48.5)	15 (45.4)	27 (45.0)	
Widowed	0 (0)	0 (0)	2 (3.3)	
Cohabiting	1 (2.8)	1 (3.0)	0 (0)	
**Ethnicity**, ***n*** **(%)**				
Caucasian	26 (74.2)	22 (66.6)	45 (75.0)	20.9, 0.02
African American	3 (8.57)	10 (30.3)	12 (20.0)	
Hispanic	6 (17.1)	1 (3.03)	3 (4.86)	
**Drinking Outcomes**				
ADS	20.25 (8.18)	22.96 (9.03)	18.62 (8.68)	0.78, 0.46
% Number of drinking days	60.38 (35.67)	59.82 (35.59)	60.60 (35.5)	0.05, 0.99
% Heavy drinking days	57.93 (35.49)	47.77 (36.84)	58.20 (36.99)	0.93, 0.39
Drinks per drinking days	16.57 (10.74)	16.27 (16.23)	16.32 (11.45)	0.006, 0.99
**Craving Outcomes**				
OCDS (total scores)	18.54 (11.13)	17.67 (11.96)	22.03 (13.31)	1.55, 0.22
**Depression Outcomes**				
HAMD (17 item total)	6.56 (3.83)	8.00 (4.31)	10.25 (5.49)	6.47, 0.002

### Drinking Outcomes

In the overall sample regardless of diagnostic group, there were significant reductions in percent of drinking days [*F*_(121, 1)_ = 252.12, *p* < 0.001], percent heavy drinking days [*F*_(121, 1)_ = 186.04, *p* < 0.001], and drinks per drinking days [*F*_(121, 1)_ = 58.75, *p* < 0.001] over time. However, there were no statistically significant differences between desipramine and paroxetine or significant interaction effects of time and desipramine/paroxetine for all drinking outcomes. Similarly, when comparing naltrexone vs. placebo there was an overall decrease in all three drinking outcomes over time (*p* < 0.001), and no statistically significant differences between naltrexone and placebo (*p* > 0.5) or significant interaction effects of time and medication for all drinking outcomes (*p* > 0.4) (see Table 2).

**Table 2:** Drinking, craving, and depression outcomes at beginning and end of treatment.

	**Desipramine**	**Paroxetine**	**Time**	**Naltrexone**	**Placebo**	**Time**
	**Mean (SE)**	**Mean (SE)**	***F, p***	**Mean (SE)**	**Mean (SE)**	***F, p***
**Drinking Outcomes**						
*% Number of drinking days*						
Pre	55.6 (5.03)	62.58 (4.48)	218.49, 0.00	62.60 (4.70)	58.14 (4.82)	226.39, 0.00
Post	7.62 (2.1)	9.36 (1.92)		9.32 (2.01)	7.31 (2.06)	
*% Heavy drinking days*						
Pre	50.3 (5.19)	56.84 (4.62)	156.8, 0.00	54.10 (4.85)	54.99 (4.97)	162.89. 0.00
Post	7.09 (2.38)	9.29 (2.12)		8.55 (2.24)	6.95 (2.29)	
*Drinks per drinking days*						
Pre	17.03 (1.81)	15.69 (1.61)	53.57, 0.00	16.34 (1.66)	16.74 (1.70)	57.71, 0.00
Post	4.19 (1.82)	7.55 (1.62)		6.01 (1.71)	5.15 (1.75)	
**Craving Outcomes**						
*OCDS (total scores)*						
Pre	19.00 (1.76)	19.92 (1.57)	8.84, 0.00	19.93 (1.56)	19.94 (1.63)	10.1, 0.00
Post	8.08 (1.55)	8.66 (1.40)		7.18 (1.43)	8.44 (1.37)	
**Depression Outcomes**						
*HAM-D (17 items)*						
Pre	11.72 (0.74)	11.66 (0.65)	7.99, 0.00	11.89 (0.69)	11.26 (0.69)	8.16, 0.00
Post	8.18 (0.83)	7.61 (0.73)		7.81 (0.75)	7.56 (0.74)	

*HAM-D, Hamilton Depression Rating Scale; OCDS, Obsessive Compulsive Drinking Scale*.

**Figure 2:**
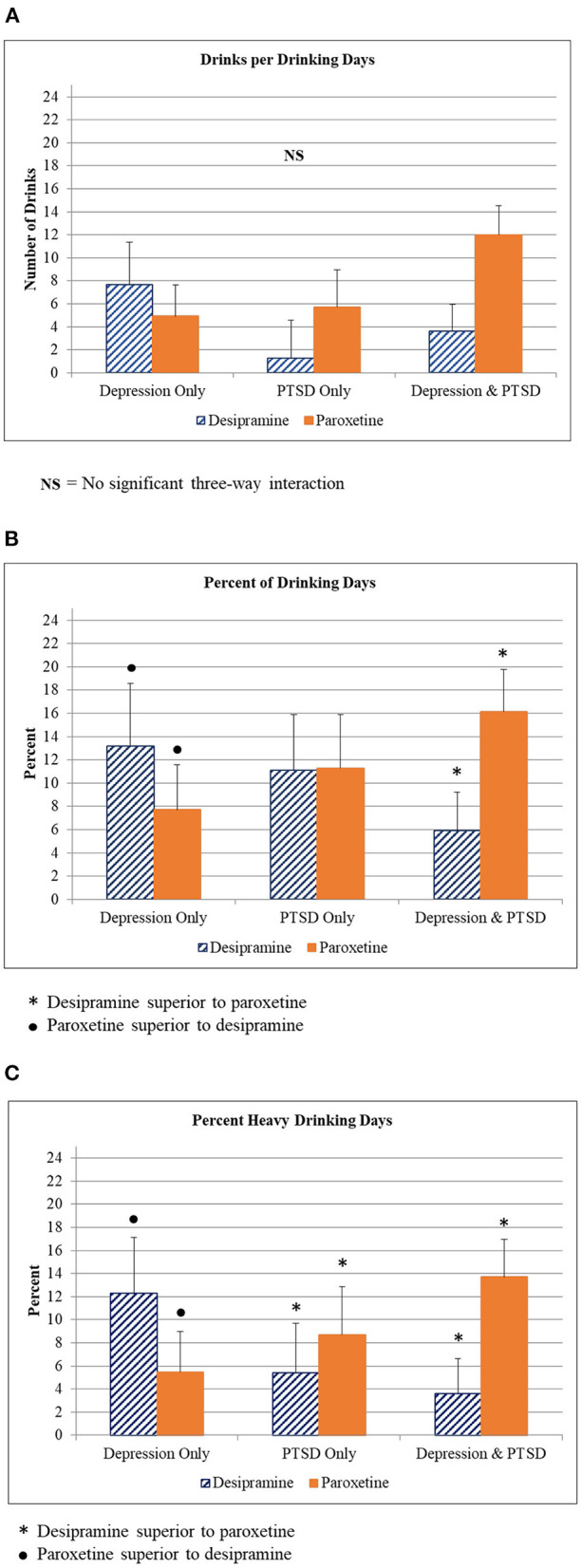
Alcohol use outcomes by diagnostic group. **(A)** Drinks per drinking day for participants in each diagnostic group (Depression only, PTSD only, Depression + PTSD) who were given either desipramine or paroxetine. **(B)** Percent of drinking day for participants in each diagnostic group (Depression only, PTSD only, Depression + PTSD) who were given either desipramine or paroxetine. Symbols indicate statistically significant differences between medications in each diagnostic group. **(C)** Percent heavy drinking day for participants in each diagnostic group (Depression only, PTSD only, Depression + PTSD) who were given either desipramine or paroxetine. Symbols indicate statistically significant differences between medications in each diagnostic group.

When diagnostic group was included in the model there were significant interactions between time, diagnostic group, and medication (desipramine vs. paroxetine) for percent heavy drinking days [*F*_(117, 2)_ = 3.431, *p* = 0.036], and percent drinking days [*F*_(117, 2)_ = 3.269, *p* = 0.04]. For the MDD group, those randomized to paroxetine showed better drinking outcomes relative to those on desipramine. This trend reversed in the PTSD group and comorbid group, which showed better drinking outcomes in the desipramine arm compared to the paroxetine arm (see Figure 2).

There were no statistically significant differences between naltrexone vs. placebo or interactions between time, diagnostic group, and naltrexone vs. placebo in drinking outcomes.

### Craving Outcomes

**Figure 3:**
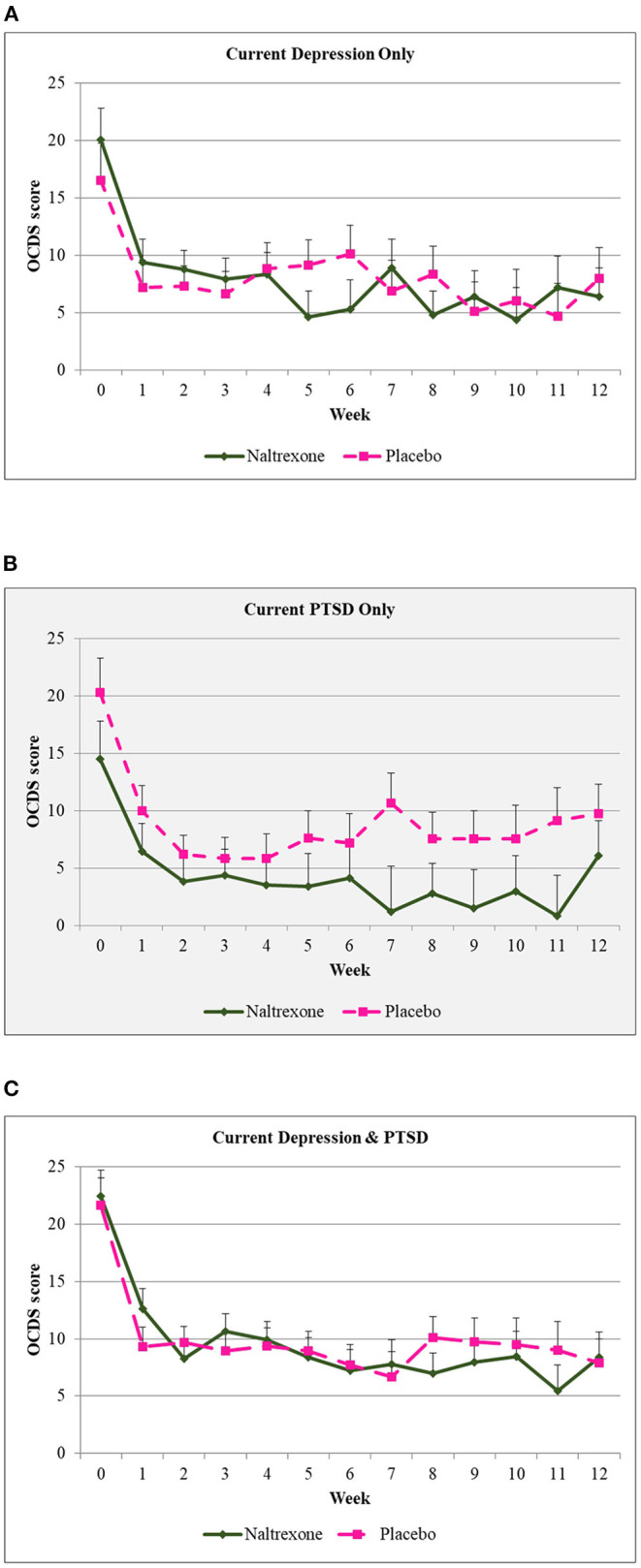
Obsessive Compulsive Drinking Scale scores over time by diagnostic group. **(A)** Craving scores over 12 weeks of treatment for those diagnosed with depression only who were given either naltrexone or placebo. **(B)** Craving scores over 12 weeks of treatment for those diagnosed with PTSD only who were given either naltrexone or placebo. Shaded background indicates that this group had a significantly lower scores on naltrexone when compared to placebo. **(C)** Craving scores over 12 weeks of treatment for those diagnosed with depression + PTSD only who were given either naltrexone or placebo.

In the overall sample, there was a significant decrease in total OCDS scores over time [*F*_(169.4, 12)_ = 9.04, *p* < 0.001]. When diagnostic group was included in the model there were significant main effects for medication (*p* = 0.02), diagnostic group (*p* = 0.000), and time (*p* = 0.000). There was a significant interaction between medication (naltrexone vs. placebo) and diagnostic group [*F*_(886.2, 2)_ = 6.28, *p* = 0.002) indicating that for the PTSD group, naltrexone showed significantly greater reduction in total OCDS scores compared to placebo. For the MDD group and the comorbid group, there were no significant differences observed (see Figure 3).

There were no statistically significant differences between desipramine and paroxetine or interactions between time, diagnostic group, and medication in total OCDS scores.

### Depressive Symptoms

In the overall sample, there was a significant decrease in HAM-D scores over time [*F*_(159, 7)_ = 7.993, *p* < 0.001]. Paroxetine demonstrated significantly greater reduction compared to desipramine [*F*_(546.1, 1)_ = 6.8, *p* = 0.009]. Interaction effect between paroxetine/desipramine and time was not statistically significant [*F*_(159.1, 7)_ = 0.506, *p* = 0.829]. There was no statistically significant difference in depressive symptom outcomes between naltrexone vs. placebo (*F* = 0.064, *p* = 0.81).

**Figure 4:**
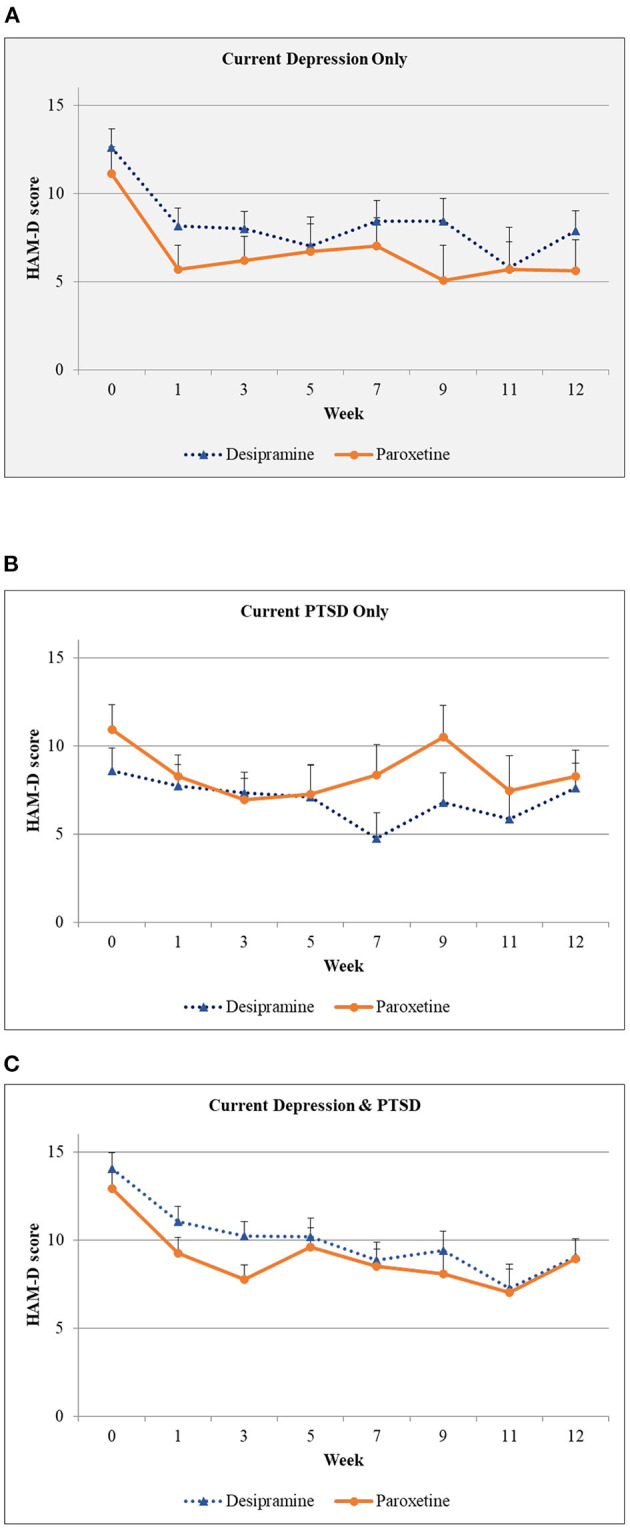
Hamilton Depression Scale scores over time by diagnostic group. **(A)** Depression scores over 12 weeks of treatment for those diagnosed with depression only who were given either desipramine or paroxetine. Shaded background indicates that this group had a significantly lower depression scores on paroxetine when compared to desipramine. **(B)** Depression scores over 12 weeks of treatment for those diagnosed with PTSD only who were given either desipramine or paroxetine. **(C)** Depression scores over 12 weeks of treatment for those diagnosed with depression + PTSD who were given either desipramine or paroxetine.

Figure 4 presents the trajectories of HAM-D scores by treatment and diagnostic groups. When diagnostic group was included in the model there was a significant interaction of medication (paroxetine/desipramine) with diagnostic group [*F*_(488.7, 2)_ = 5.012, *p* = 0.007] with paroxetine showing greater reduction compared to desipramine in HAM-D scores in the MDD group. However, there was no significant three-way interaction [*F*_(153.4, 14)_ = 0.284, *p* = 0.995].

As expected, there was no statistically significant difference in depressive symptom outcomes between diagnostic group for those that were randomized to either naltrexone or placebo.

## Discussion

In this randomized clinical trial of 128 veterans with AUD and co-occurring MDD and/or PTSD, we found different responses to antidepressant medication in drinking outcomes by comorbidity; paroxetine demonstrated better drinking results than desipramine for those with co-occurring MDD, whereas desipramine showed better drinking results relative to paroxetine for individuals with PTSD, even in the case of concurrent major depression (MD). With regards to craving, naltrexone showed greater superiority over placebo, and this difference was driven by the response in the PTSD group. For depressive symptoms overall, paroxetine was statistically superior to desipramine in reducing depressive symptoms in the full sample. There was no clinical advantage of adding naltrexone compared to placebo.

The most notable finding of our study is the difference in response of drinking outcomes to desipramine vs. paroxetine by comorbidity profile. The findings extend the results from our previous study (14). Consistent with that study, there was superiority of desipramine over paroxetine in alcohol use outcomes among those with PTSD. However, when there is MDD without co-occurring PTSD diagnosis, paroxetine reduced alcohol use compared to desipramine. Desipramine has been tested previously in MDD with co-occurring AUD in a placebo-controlled RCT of 71 patients with AUD (18). Desipramine reduced alcohol relapse compared to placebo in patients with co-occurring depression and AUD, but not in those without depression (18). Since the present study did not have a placebo antidepressant, it is unknown whether desipramine would fare better than placebo and whether there the difference between desipramine and paroxetine is clinically meaningful. There is interest in using noradrenergic agents, such as prazosin (39, 40) and doxazosin (41, 42) to treat AUD alone and as it co-occurs with PTSD (14, 43). However, there are no studies to our knowledge on whether these agents are also effective with co-occurring MDD. Although noradrenergic mechanisms are implicated in both MDD and PTSD (26, 27), this study suggests that effectiveness of some medications used for AUD may yield different responses among those with co-occurring MDD when it occurs without PTSD. This would be an important area of further study, given how commonly MDD occurs in AUD.

Adding naltrexone to an antidepressant did not demonstrate statistical significance in reduction of drinking outcomes over placebo. One possible explanation of this finding is that a “floor effect” may have affected the results of this study (44). Further, the effect of naltrexone may not have been detected due to lack of power. However, naltrexone significantly decreased cravings compared to placebo in the PTSD group which is consistent with the primary paper (14), however, this did not demonstrate differences in the comorbid and MDD groups.

Future studies investigating the role of comorbidity in response to antidepressants should also consider the differences in responses by clinical subtypes of AUD. For example, age of onset has been found to influence outcomes with serotonergic agents in terms of depression (45, 46) and similarly, presence of comorbidity also has been shown to be important (20). Consistent with this, in a study of PTSD and AUD, late onset AUD and early onset PTSD was associated with better outcomes while those with more severe AUD and later-onset PTSD who were treated with sertraline showed poorer drinking outcomes relative to placebo (21).

The finding that paroxetine was superior to desipramine in reducing depressive symptoms regardless of diagnostic group has several caveats. Further examination suggests this finding was driven primarily by the subsample of those with MDD alone; our previous study in those with PTSD, showed no difference in paroxetine or desipramine in reducing symptoms of PTSD or depressive symptoms. Second, it should be noted that these differences were small and may not have been clinically significant. Baseline depression scores were in the mild range and there may have been a floor effect (44). There was no placebo comparison, so it is unknown whether this was a true medication effect. It is also plausible that reduction of alcohol use may have partially mediated the reduction of depressive symptoms. The comparative effectiveness between serotonergic and noradrenergic antidepressants in reducing depressive symptoms has been studied in numerous trials in the depression literature (47). In a meta-analysis of 15 head-to-head RCTs comparing SSRIs and SNRIs showed that SNRIs had statistical significance over SSRIs in treating MDD (47); these studies did not include participants with AUD. In the AUD literature, a recent network meta-analysis of 68 RCTs consisting of 5,890 patients with AUD comparing different classes of antidepressants including SSRIs (e.g., paroxetine, citalopram, escitalopram, fluoxetine, and sertraline), NRIs (e.g., venlafaxine and viloxazine), and tricyclic antidepressants (e.g., desipramine, imipramine, amitriptyline) showed that NRIs demonstrated the best efficacy and were superior to SSRIs in reducing scores of depression scales (48). However, in that study, desipramine was categorized as a tricyclic antidepressant, which did not show any statistically significant difference in both depression and AUD outcomes when compared to SSRIs (including paroxetine) (48).

This study has several limitations. First, given the nature of the parent trial, the overall HAM-D scores were low on average, even though all participants met criteria for MDD. Second, we were not able to look into the clinical subtypes of AUD, which have been suggested to impact treatment response of alcohol use outcomes to antidepressants in previous trials of co-occurring AUD and PTSD (21). Third, there were large drop outs in both groups (54% for desipramine vs. 47% for paroxetine). An attrition analysis was not conducted given that the attrition rates were similar in both groups. Lastly, the study sample was predominantly male which limits its generalizability to women.

Notwithstanding these limitations, this study provides preliminary evidence on how different comorbidities may influence clinical practices in individuals with AUD and co-occurring psychiatric disorders. Clinical implications of these findings are that when a patient presents with AUD and MDD, paroxetine may have an advantage in addressing both depressive symptoms and alcohol use outcomes. In addition to the findings of the primary study showing that for patients with co-occurring AUD and PTSD, desipramine may have an advantage in alcohol use outcomes, it further suggests that when individuals with co-occurring AUD and PTSD also meet criteria for MDD, desipramine may have even larger benefits over paroxetine.

## Data Availability Statement

The raw data supporting the conclusions of this article may be made available by the authors, upon request.

## Ethics Statement

The studies involving human participants were reviewed and approved by VA Connecticut HSS and Yale IRB. The patients/participants provided their written informed consent to participate in this study.

## Author Contributions

IP: supervised all aspects of study conduct, acted as senior author of the manuscript, and contributed to the writing of the manuscript. ER: conducted data analysis, contributed to the writing of the manuscript, and supervised the recruitment and study conduct. PN: contributed to the drafting, writing, and editing processes for the manuscript. OJ and AW: contributed to literature review and editing of the manuscript. All authors contributed to the article and approved the submitted version.

## Funding

This study was funded by the New England Mental Illness Research Education and Clinical Center (MIRECC) and the National Center for PTSD (NC-PTSD).

## Conflict of Interest

IP has served as a consultant for Alkermes and BioXcel Therapeutics and has received in-kind medication from Alkermes and BioXcel for grant-funded studies. The remaining authors declare that the research was conducted in the absence of any commercial or financial relationships that could be construed as a potential conflict of interest.

## Publisher's Note

All claims expressed in this article are solely those of the authors and do not necessarily represent those of their affiliated organizations, or those of the publisher, the editors and the reviewers. Any product that may be evaluated in this article, or claim that may be made by its manufacturer, is not guaranteed or endorsed by the publisher.
